# Supplementary material for: A natural biogenic nanozyme for scavenging superoxide radicals
Source: Nat Commun. 2024 Jan 3;15:233. doi: 10.1038/s41467-023-44463-w (PMC10764798; doi:10.1038/s41467-023-44463-w)
Supplement: Supplementary file 1 — Supplementary Information [file 41467_2023_44463_MOESM1_ESM.pdf]

Supplementary Information

A natural biogenic nanozyme for scavenging superoxide radicals

Long Ma[1,2], Jia-Jia Zheng[3], Ning Zhou[1], Ruofei Zhang[1], Long Fang[1,4], Yili Yang[5], Xingfa Gao[3], Chunying Chen[6], Xiyun Yan[1,2,7,8,*] and Kelong Fan[1,2,7,8,*]

1 CAS Engineering Laboratory for Nanozyme, Key Laboratory of Biomacromolecules (CAS), CAS Center for Excellence in Biomacromolecules, Institute of Biophysics, Chinese Academy of Sciences, Beijing, 100101, China

2 University of Chinese Academy of Sciences, Chinese Academy of Sciences, Beijing, 100408, China

3 Laboratory of Theoretical and Computational Nanoscience, National Center for Nanoscience and Technology of China, Beijing, 100190, China

4 Savaid Medical School, University of Chinese Academy of Sciences, Beijing, 100049, China

5 China Regional Research Centre, International Centre for Genetic Engineering and Biotechnology, Taizhou, Jiangsu, 225316, China

6 CAS Key Laboratory for Biomedical Effects of Nanomaterials and Nanosafety and CAS Center for Excellence in Nanoscience, National Center for Nanoscience and Technology, Beijing, 100190, China

7 Nanozyme Medical Center, School of Basic Medical Sciences, Zhengzhou University, Zhengzhou, Henan, 450052, China

8 Nanozyme Laboratory in Zhongyuan, Zhengzhou, Henan, 451163, China

*Corresponding authors: E-mail: yanxy@ibp.ac.cn (X.Y.); fankelong@ibp.ac.cn (K.F.)

The Supplementary Information includes:

16 Supplementary Figures

2 Supplementary Tables

Supplementary Figures

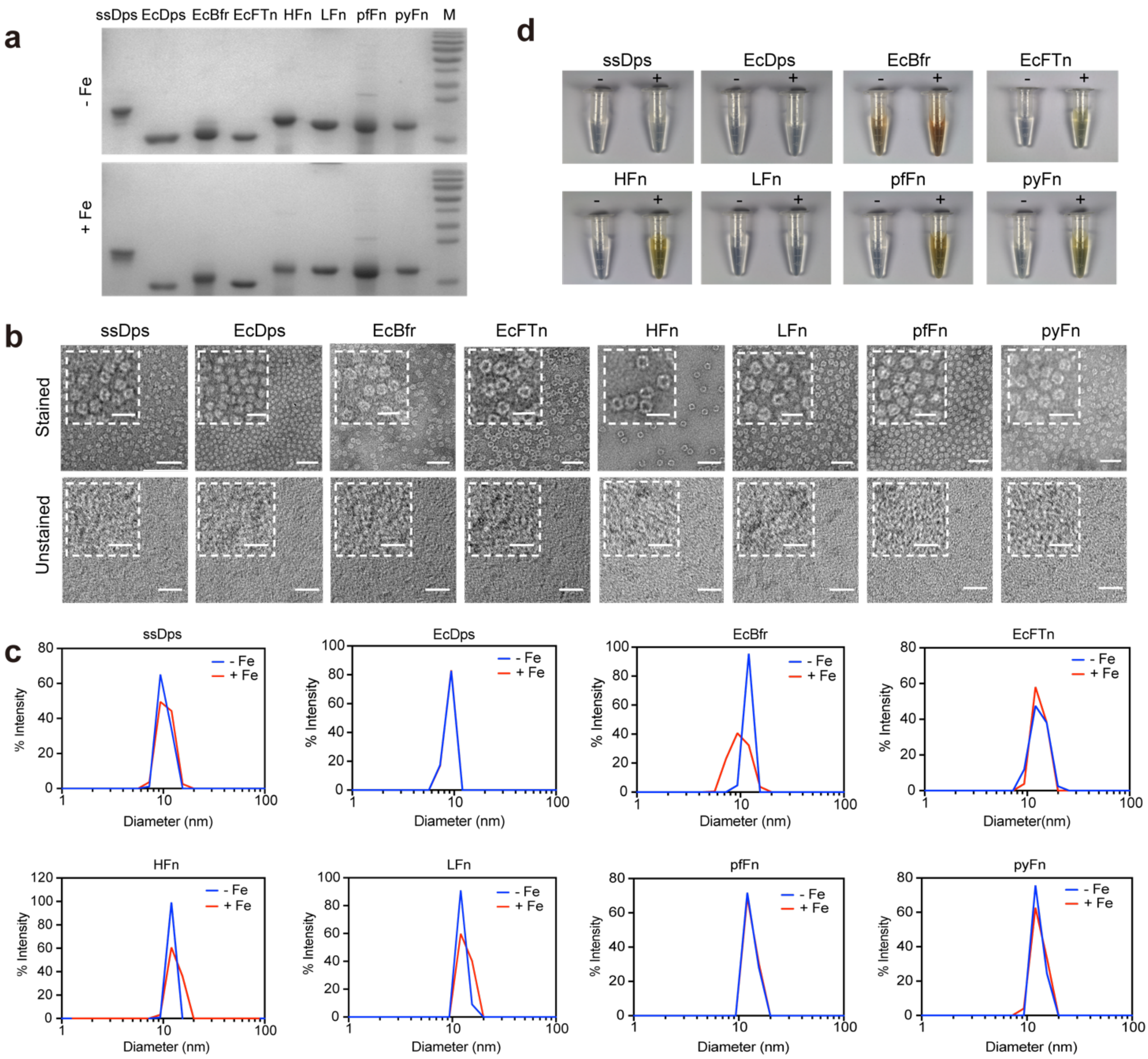

Supplementary Figure 1. Characterization of the ferritins from different species. (**a**) SDS-PAGE of ferritins purified from the basic medium (- Fe) and medium containing excess iron (+ Fe). M represents protein marker. (**b**) Negative stained and unstained TEM images of ferritins purified from the basic medium of three independent experiments with similar results; inset figures represented partially enlarged images; the scale bar referred to 50 nm; the inset scale bar referred to 20 nm. (**c**) DLS spectra of ferritins purified from the basic medium (- Fe) and medium containing excess iron (+ Fe). (**d**) Digital images of ferritins purified from the basic medium (-) and medium containing excess iron (+). Source data are provided as a Source Data file.

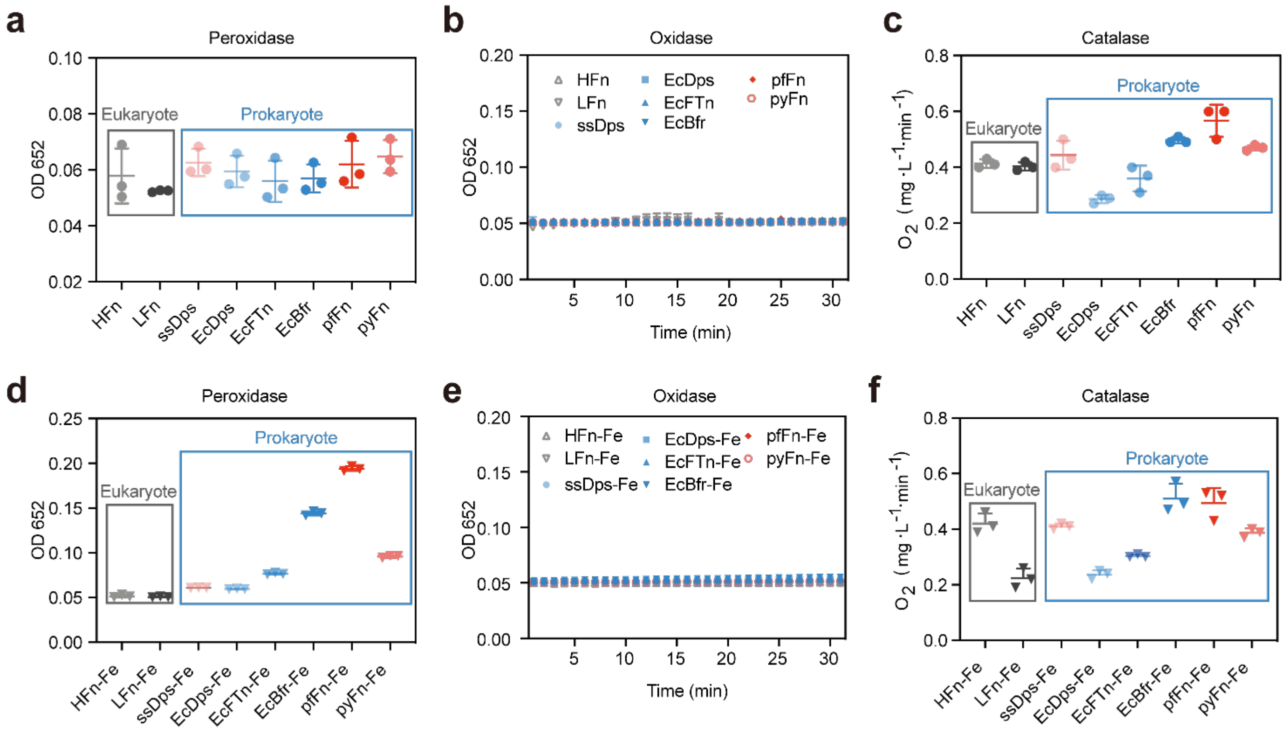

Supplementary Figure 2. The peroxidase, oxidase, and catalase-like activities of ferritins from different species. (**a**) Peroxidase-like activity of ferritins purified from the basic medium. (**b**) Oxidase-like activity of ferritins purified from the basic medium. (**c**) Catalase-like activity of ferritins purified from the basic medium. (**d**) Peroxidase-like activity of ferritins purified from medium containing excess iron. (**e**) Oxidase-like activity of ferritins purified from medium containing excess iron. (**f**) Catalase-like activity of ferritins purified from medium containing excess iron. All data are mean ± SD (*n* = 3 independent experiments). Source data are provided as a Source Data file.

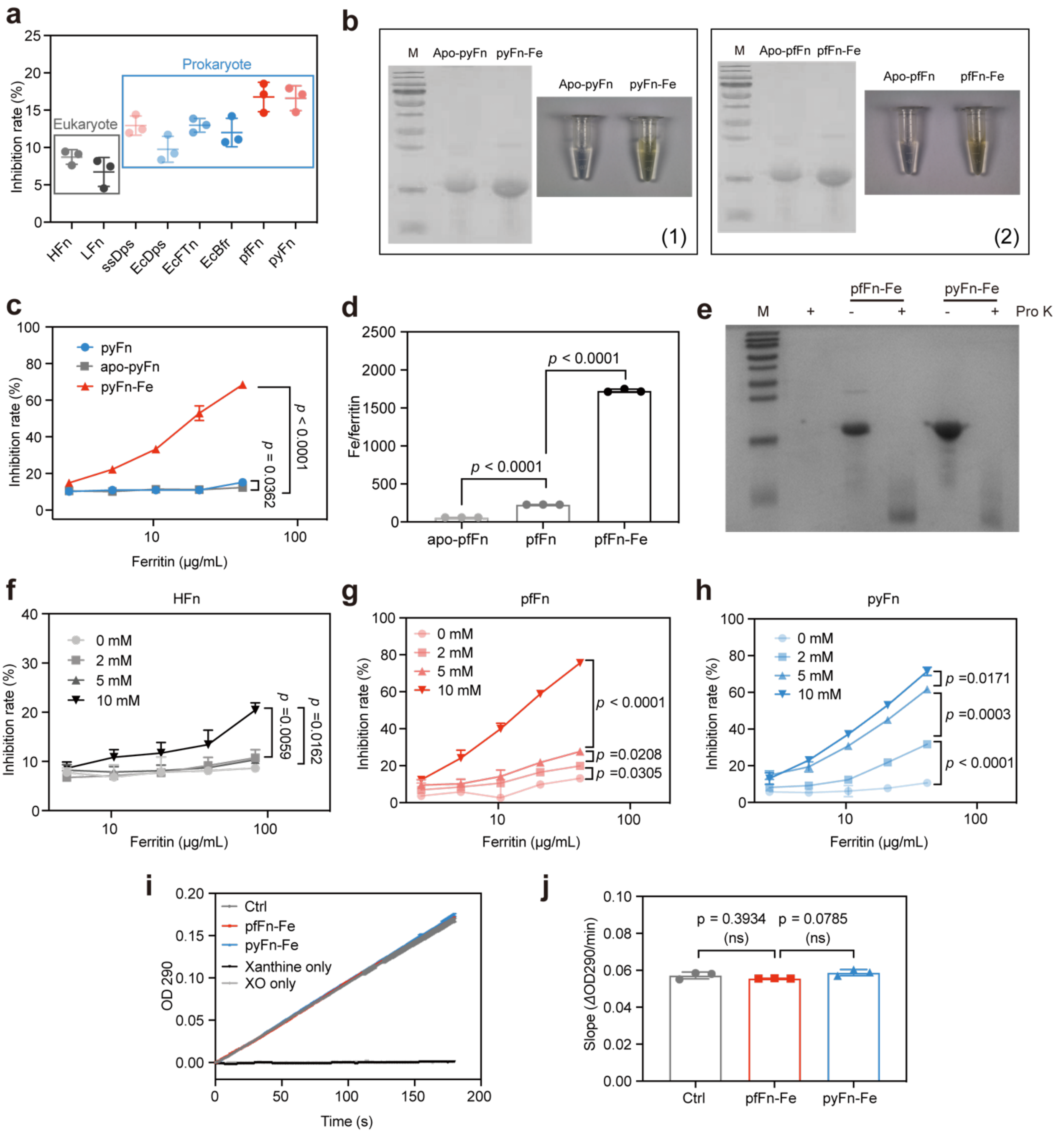

Supplementary Figure 3. SOD-like activity and characterizations of ferritins with the core or protein shell removed. (**a**) SOD-like activities of ferritins purified from the basic medium; the final concentration of the ferritins was 50 μg/mL. (**b**) SDS-PAGE and digital images of pyFn-Fe (1) and pfFn-Fe (2) before (Fn-Fe) and after the iron removal (apo-Fn). M represents protein marker. (**c**) The SOD-like activity of apo-pyFn, pyFn, and biomineralized pyFn (pyFn-Fe). (**d**) Iron content of apo-pfFn, pfFn, and biomineralized ferritins (pfFn-Fe). (**e**) SDS-PAGE of protease K digested pfFn-Fe and pyFn-Fe. M represents protein marker. (**f-h**) SOD-like activity of biomineralized ferritins with gradient iron content. (**i**) The urate generation curve of the xanthine-xanthine oxidase system with the ferritin concentration at 100 μg/mL; Ctrl referred to the xanthine oxidase/xanthine without the ferritin addition. (**j**) The urate generation slope of the xanthine-xanthine oxidase system. All data are presented as mean

± SD ($n = 3$ independent experiments); the significant differences were evaluated by Two-way ANOVA with post-hoc Tukey HSD test for **c**, **f**, **g**, **h**, and by One-way ANOVA with post-hoc Tukey HSD test for **d** & **j**; ns: not significant, $p > 0.05$. Source data are provided as a Source Data file.

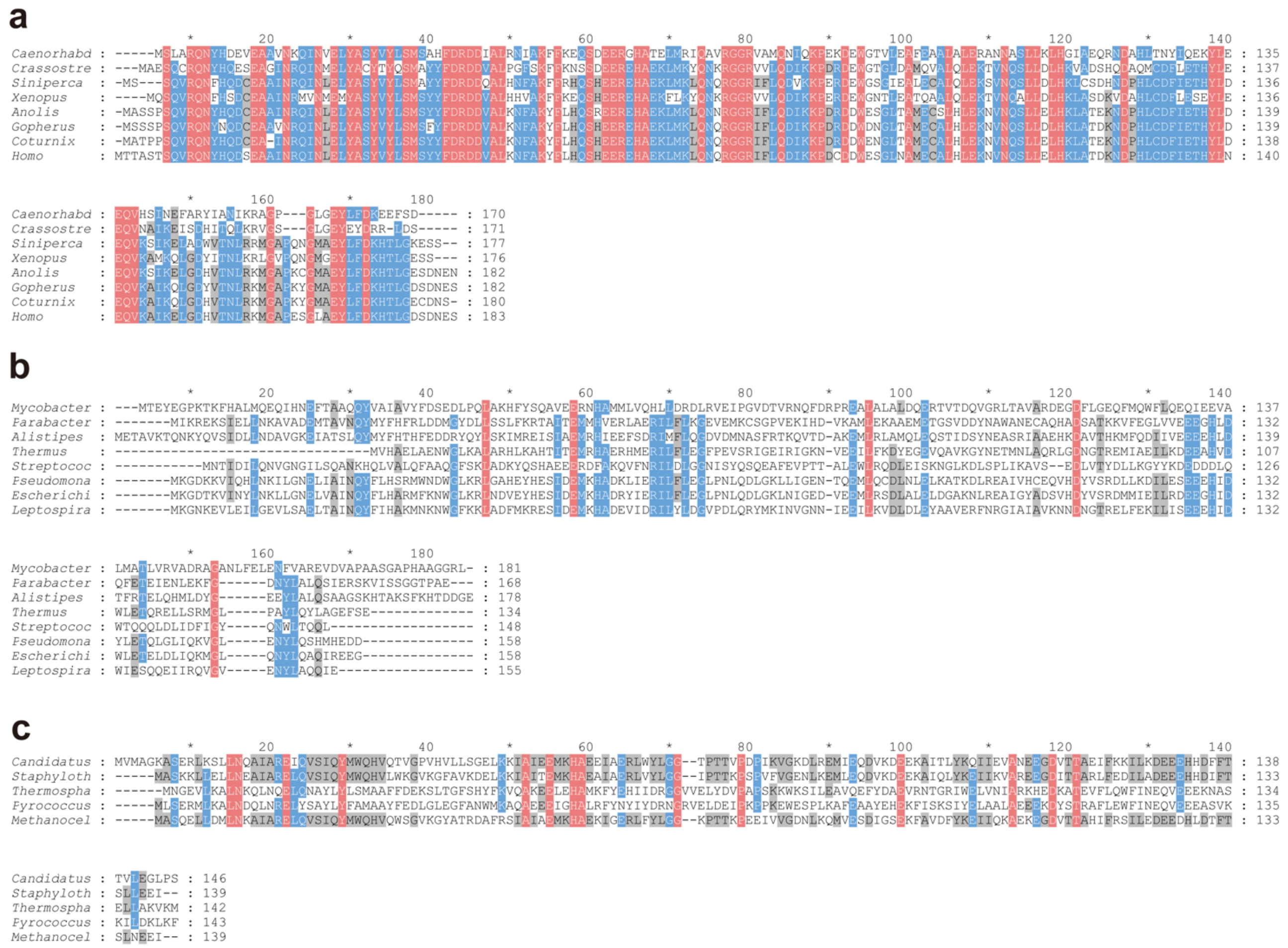

Supplementary Figure 4. Sequence alignment of the ferritins from different species. (**a**) Multiple-sequence alignment of the different animal ferritin sequences from different phyla (*Caenorhabditis elegans*, *Crassostrea gigas*, *Siniperca chuatsi*, *Xenopus laevis*, *Anolis carolinensis*, *Gopherus evgoodei*, *Coturnix japonica*, *Homo sapiens*). (**b**) Multiple-sequence alignment of different bacteria ferritins from different phyla (*Mycobacterium tuberculosis*, *Parabacteroides goldsteinii*, *Alistipes onderdonkii*, *Thermus scotoductus*, *Streptococcus equinus*, *Pseudomonas aeruginosa*, *Escherichia coli*, *Leptospira weilii*). (**c**) Multiple-sequence alignment of different archaea ferritins from different phyla (*Candidatus Korarchaeum*, *Staphylothermus marinus*, *Thermosphaera aggregans*, *Pyrococcus yayanosii*). The colors referred to the amino acid similarity, red: 100%, blue: 80%, gray: 60%.

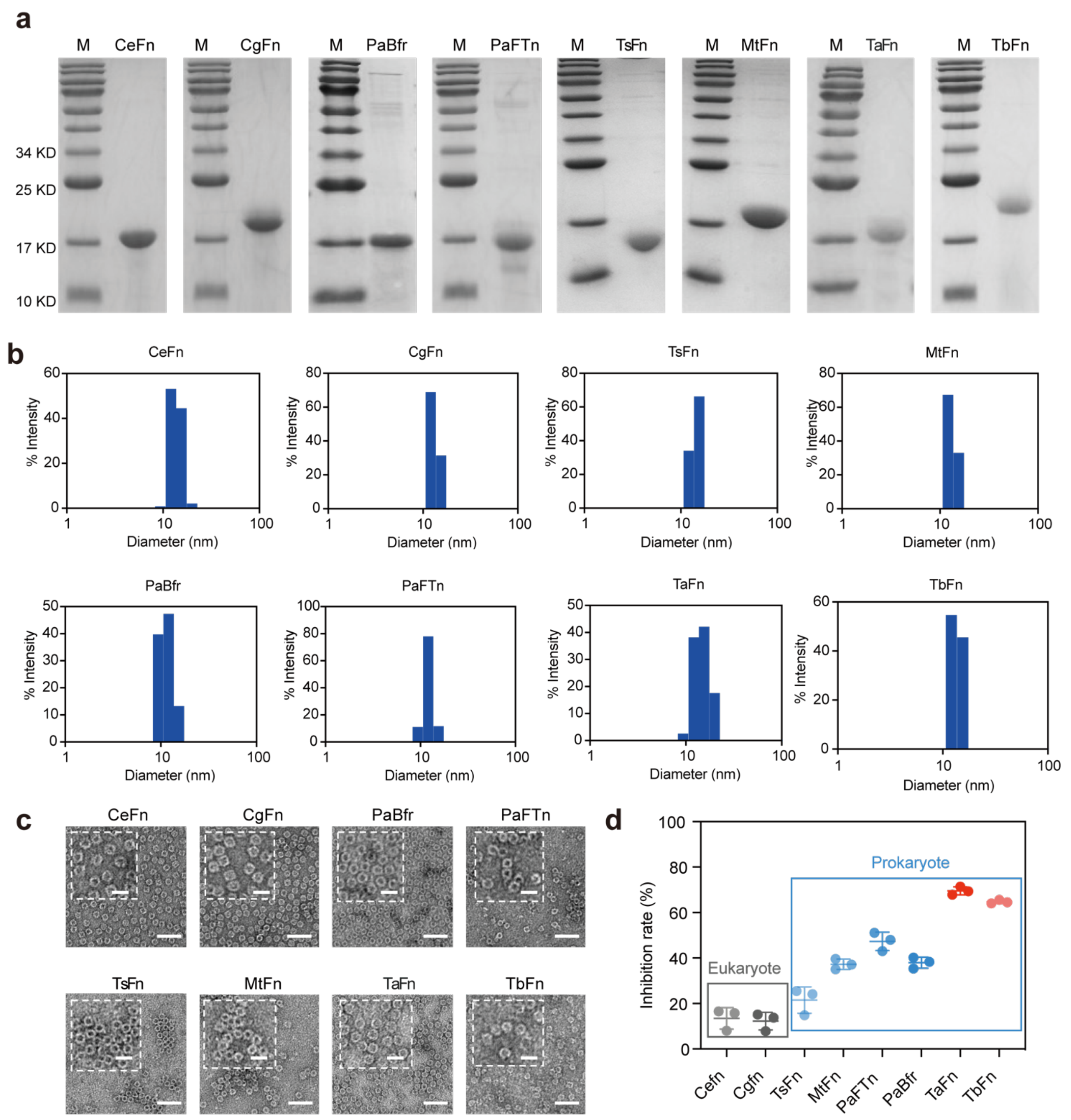

Supplementary Figure 5. Characterization and SOD-like activity of ferritins from different species. (**a**) SDS-PAGE of ferritins from different species (*Caenorhabditis elegans* (CeFn), *Crassostrea gigas* (CgFn), *Pseudomonas aeruginosa* bacterioferritin (PaBfr), *Pseudomonas aeruginosa* ferritin (PaFTn), *Mycobacterium tuberculosis* (MtFn), T*hermus scotoductus* (TsFn), *Thermosphaera aggregans* (TaFn), *Thermococcus barophilus* (TbFn)). M represents protein marker. (**b**) DLS spectra of ferritins from different species. (**c**) Negative stained TEM images of ferritins from different species of three independent experiments with similar results; inset figures represented partially enlarged images; the scale bar referred to 50 nm; inset scale bar referred to 20 nm. (**d**) SOD-like activities of ferritins from different species with the final ferritin concentration at 50 μg/mL; data are presented as mean ± SD (*n* = 3 independent experiments). Source data are provided as a Source Data file.

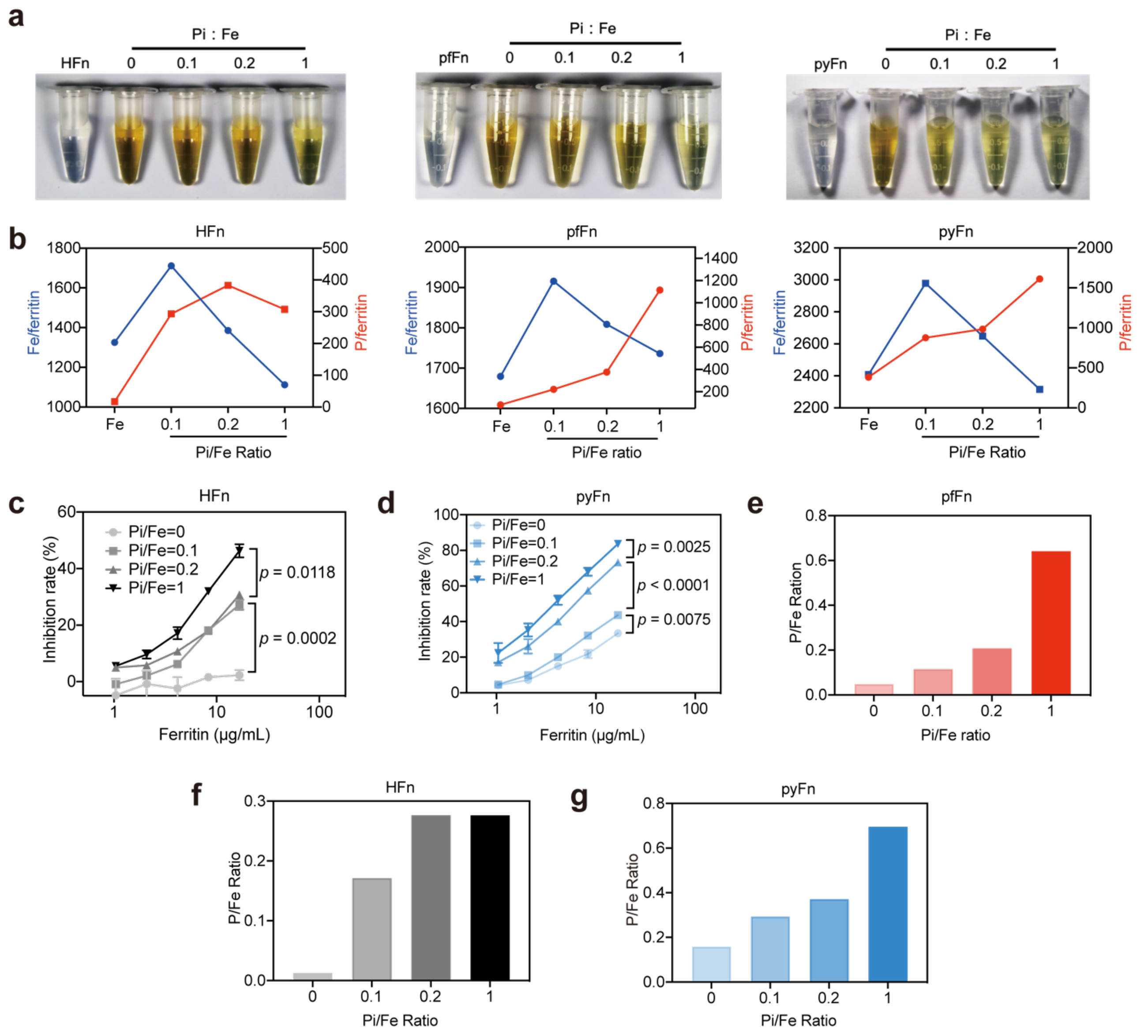

Supplementary Figure 6. Characterization of ferritin reconstituted with different phosphate/iron (Pi/Fe) ratios. (a) Digital image of the ferritins reconstituted with the iron and gradient phosphate. (**b**) Iron and phosphorus content of the ferritins reconstituted with iron and gradient phosphate; data are presented as mean ± SD (n = 3 independent experiments). (**c, d**) SOD-like activities of the HFn and pyFn reconstituted with iron and gradient phosphate; data are presented as mean ± SD (n = 3 independent experiments); the significant differences were evaluated by Two-way ANOVA with post-hoc Tukey HSD test. (**e-g**) P/Fe ratios of pfFn, HFn, pyFn reconstituted with iron and gradient phosphate. Source data are provided as a Source Data file.

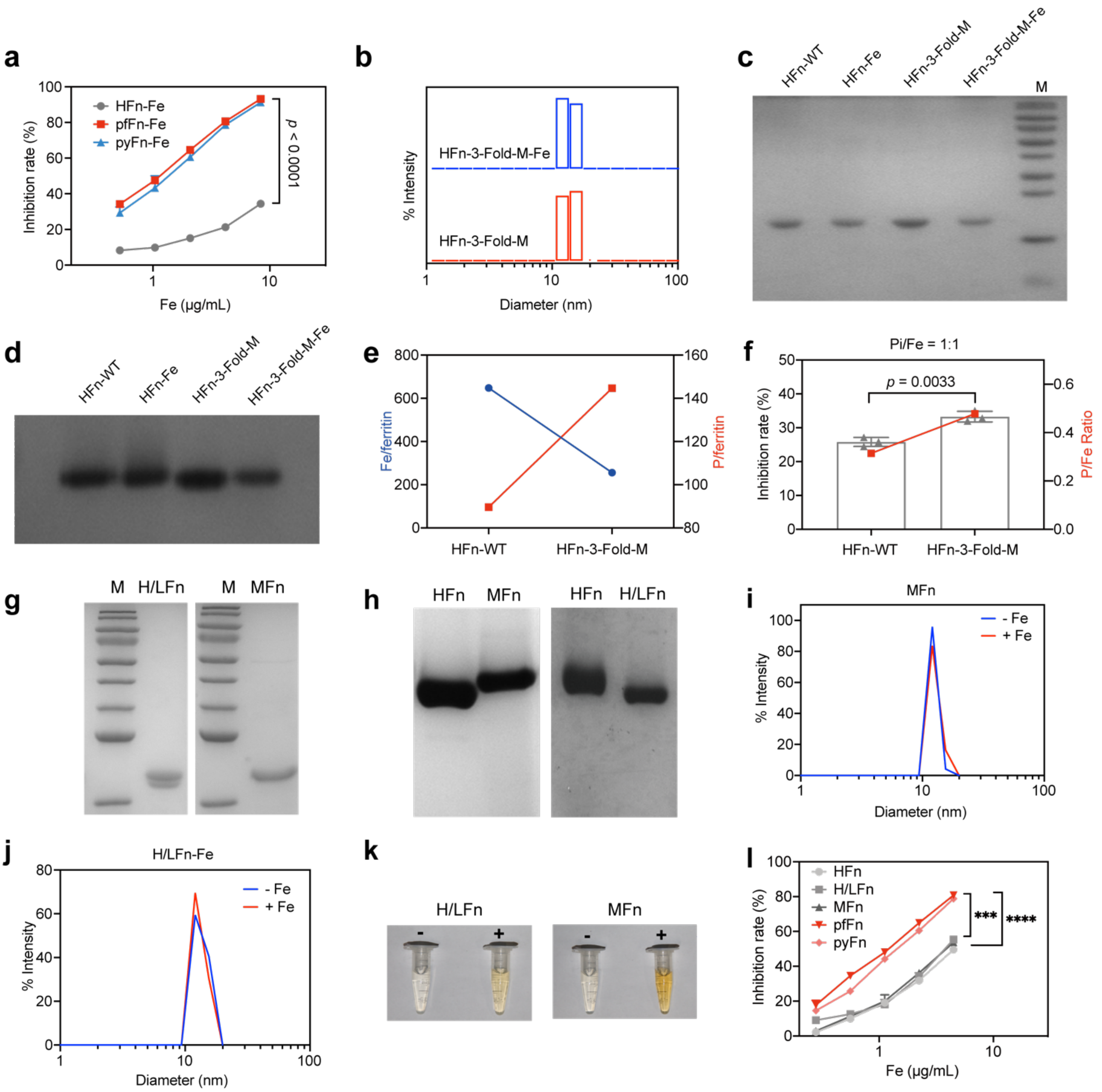

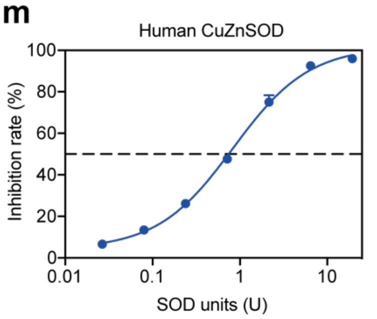

n

	IC50 (μg/mL)	SOD units (U/mg/Fe) based on SOD Kit	SOD units (U/mg/Fe) based on commercial CuZnSOD
HFn-Fe	3.775	1103.75276	924.834437
H/LFn-Fe	3.277	1271.48815	1065.37992
MFn-Fe	3.531	1180.02454	988.742566
pfFn-Fe	1.198	3478.01892	2914.23205
pyFn-Fe	1.617	2576.78829	2159.09091

Supplementary Figure 7. Characterization and SOD-like activity of the three-fold pore mutated HFn, human mitochondria ferritin, and human H/L heteropolymer ferritin. (**a**) SOD-like activity of biomineralized HFn, pfFn and pyFn in the same total iron content; data are presented as mean ± SD (n = 3 independent experiments); the significant differences were evaluated by Two-way ANOVA with post-hoc Tukey HSD test. (**b**) DLS spectra, (**c**) SDS-PAGE and (**d**) Native gel of wild type and three-

fold pore mutated HFn; HFn-3-Fold-M-Fe referred to biomineralized HFn three-fold pore mutant. M represents protein marker. (**e**) Iron and phosphorus content of the biomineralized HFn and HFn mutant. (**f**) SOD-like activity and Iron, phosphorus content of the reconstituted HFn and HFn mutant; data are presented as mean ± SD (n = 3 independent experiments); the significant differences were evaluated by Two-tailed unpaired Student's t-test. (**g, h**) SDS-PAGE and Native gel of human mitochondria ferritin and human H/L heteropolymer ferritin. M represents protein marker. (**i, j**) DLS spectrum of the human mitochondrial ferritin and H/L heteropolymer ferritin. (**k**) Digital images of human mitochondrial ferritin and H/L heteropolymer ferritin solution. (**l**) SOD activities of the reconstituted HFn, H/LFn, MFn, pfFn, pyFn in the same total iron content; data are presented as mean ± SD (n = 3 independent experiments); the significant differences were evaluated by Two-way ANOVA with post-hoc Tukey HSD test: p value between pfFn or pyFn with the HFn or H/LFn was < 0.0001; between pfFn and MFn was 0.001; between pyFn and MFn was 0.0006. (**m**) Inhibition rate on the reaction between the superoxide and WST-1 of commercial Human CuZnSOD; data are presented as mean ± SD (n = 3 independent experiments). (**n**) Calculated SOD units of reconstituted HFn-Fe, H/LFn-Fe, MFn-Fe, pfFn-fe, pyFn-Fe based on the method of SOD assay kit or the commercial CuZnSOD. Source data are provided as a Source Data file.

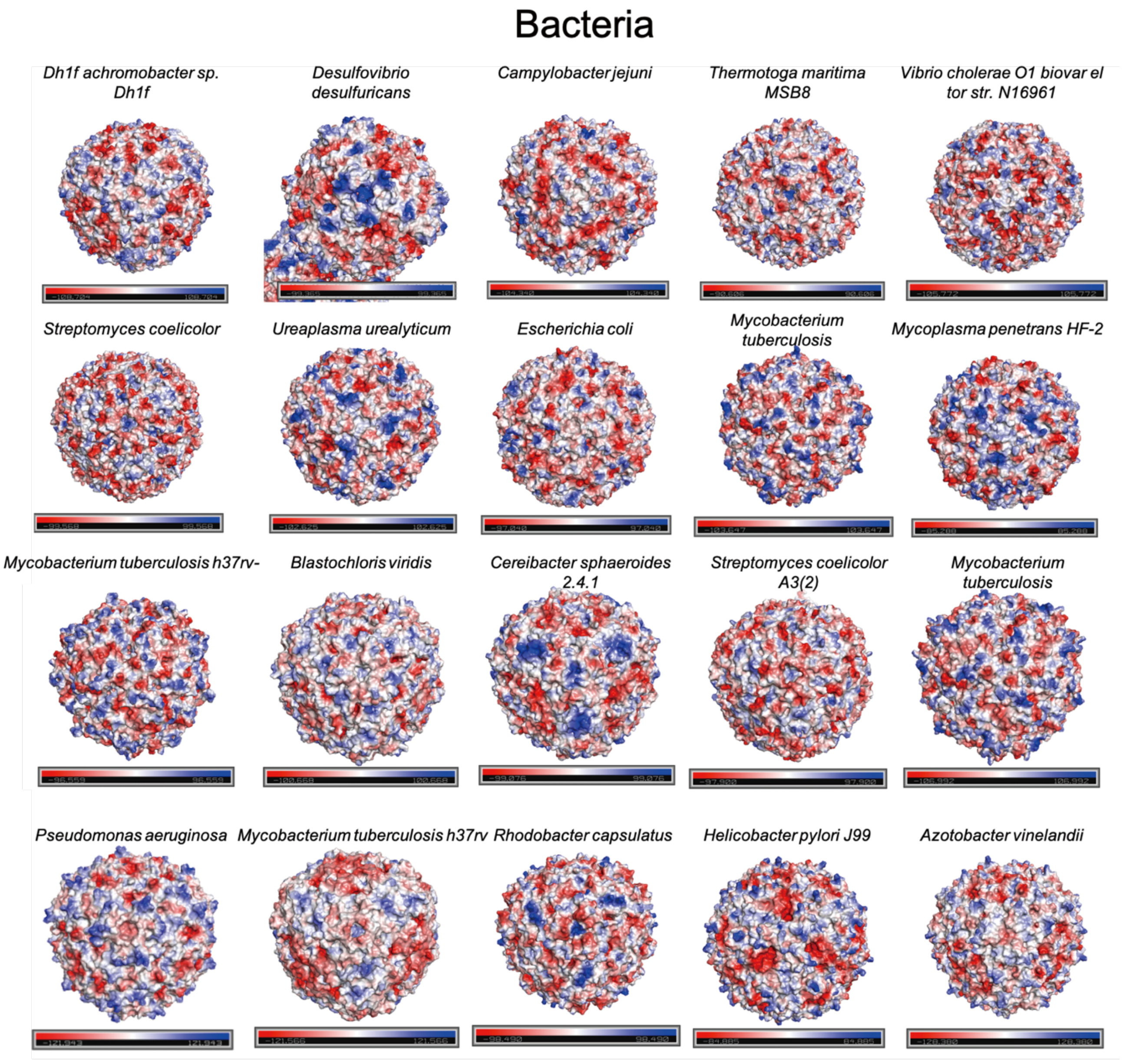

Supplementary Figure 8. The surface potential of 20 different bacteria ferritins from the PDB database. (PDB ID (from left to right): first line: 5ZUR, 1NF4, 3KWO, 1Z4A, 3QZ3; second line: 6K3O, 7VP8, 1EUM, 3OJ5, 7DIE; Third line: 7O6E, 4AM2, 3GVY, 5XX9, 3UNO; fourth line: 3R2I, 3QB9, 1JGC, 3EGM, 1SOF).

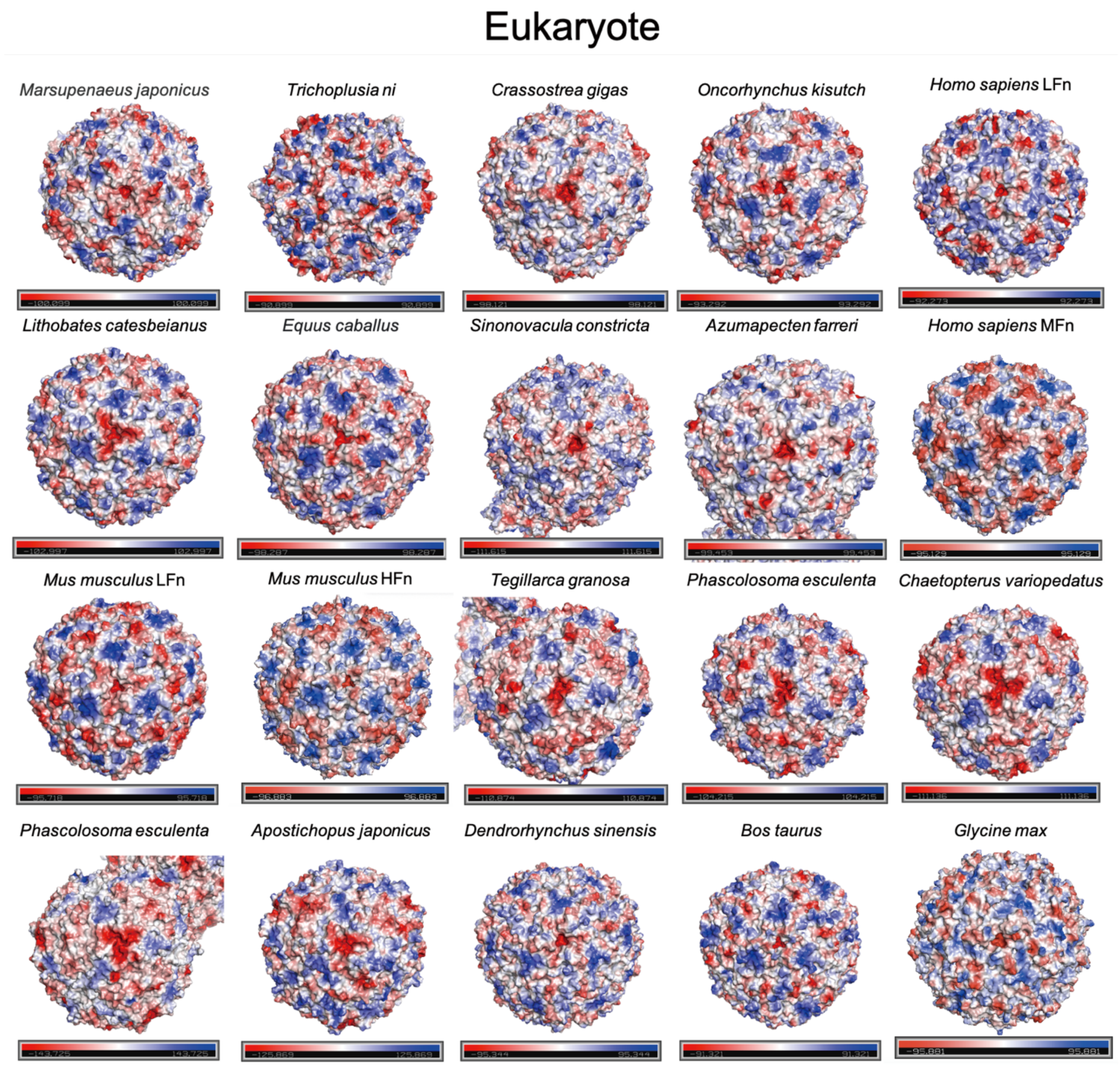

Supplementary Figure 9. The surface potential of 20 different eukaryote ferritins from the PDB database. (PDB ID (from left to right): first line: 6A4U, 1Z6O, 6LIJ, 6LK9, 2FG4; second line: 4QLH, 1IER, 6LQ5, 7VT2, 1R03; third line: 1H96, 6S61, 6L55, 6LPE, 5WPN; fourth line: 6LPD, 7VHR, 7EMK, 7U5L, 3A68).

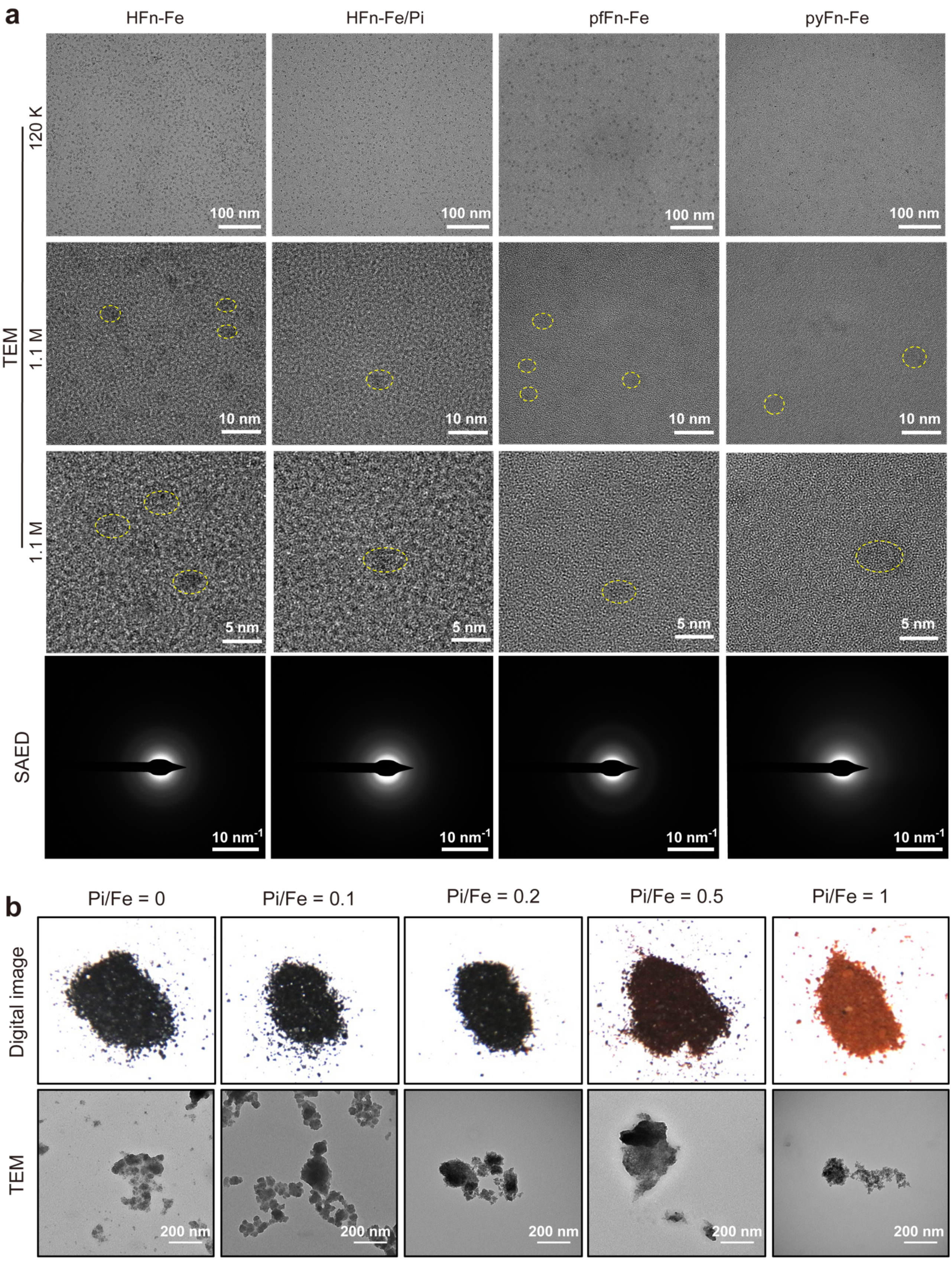

Supplementary Figure 10. Characterization of the iron core in different ferritins and phosphate-doped ferrihydrite. (**a**) TEM and SAED images of the iron core in reconstituted HFn, HFn

reconstituted with iron and phosphate, pfFn and pyFn. (**b**) Digital and TEM images of the 2-line ferrihydrite with the gradient phosphate doped.

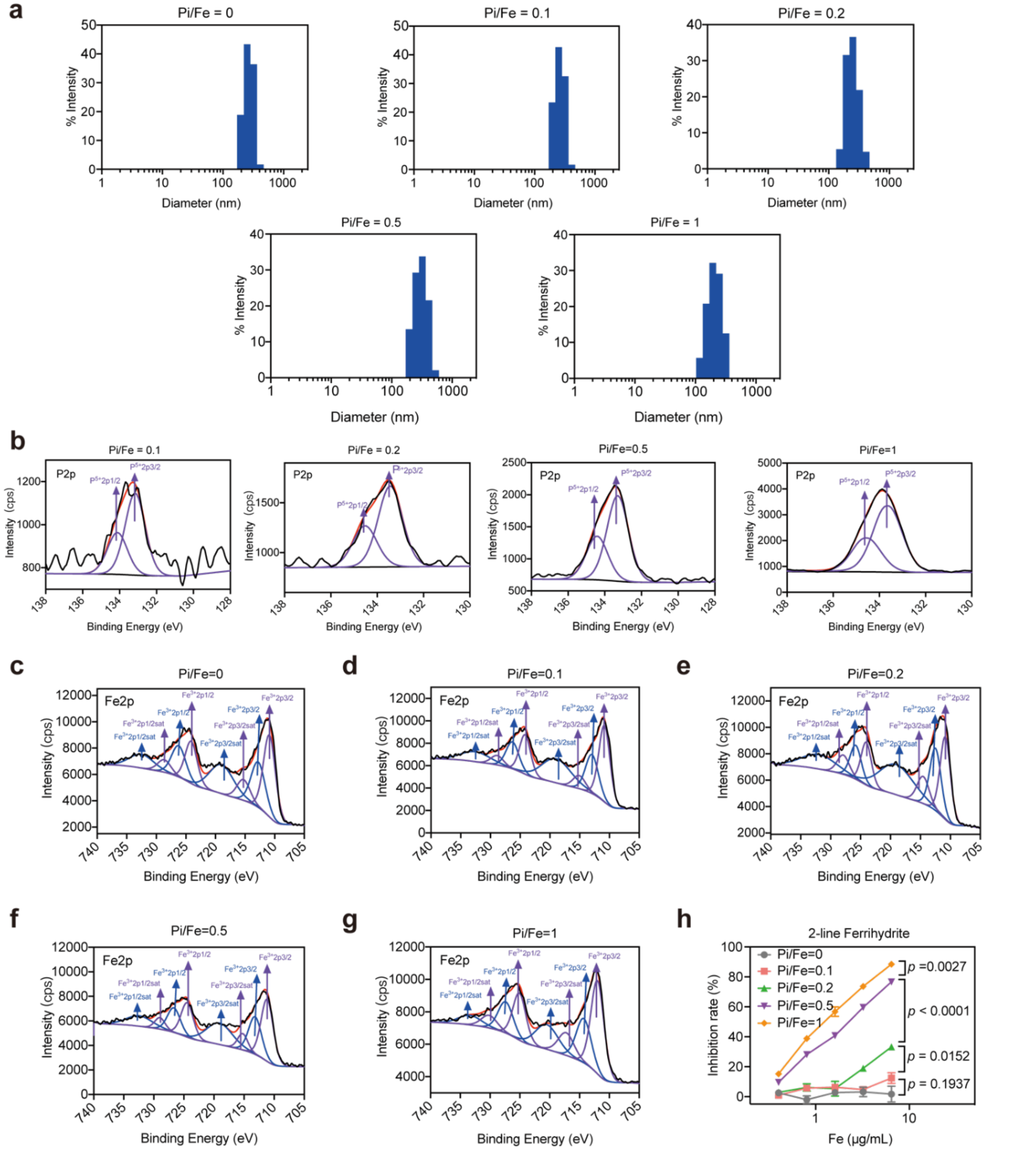

Supplementary Figure 11. Characterization of 2-line ferrihydrite doped with the gradient phosphate. (**a**) DLS spectra of 2-line ferrihydrite and the phosphate-doped 2-line ferrihydrite. (**b**) XPS fitted curve of the phosphorus element in 2-line ferrihydrite doped with the gradient phosphate. (**c-g**) XPS fitted curve of the iron element in 2-line ferrihydrite doped with the gradient phosphate. (**h**) SOD-like activity of 2-line ferrihydrite doped with the gradient phosphate in the equal iron content; data are presented as mean ± SD (n = 3 independent experiments); the significant differences were evaluated by Two-way ANOVA with post-hoc Tukey HSD test. Source data are provided as a Source Data file.

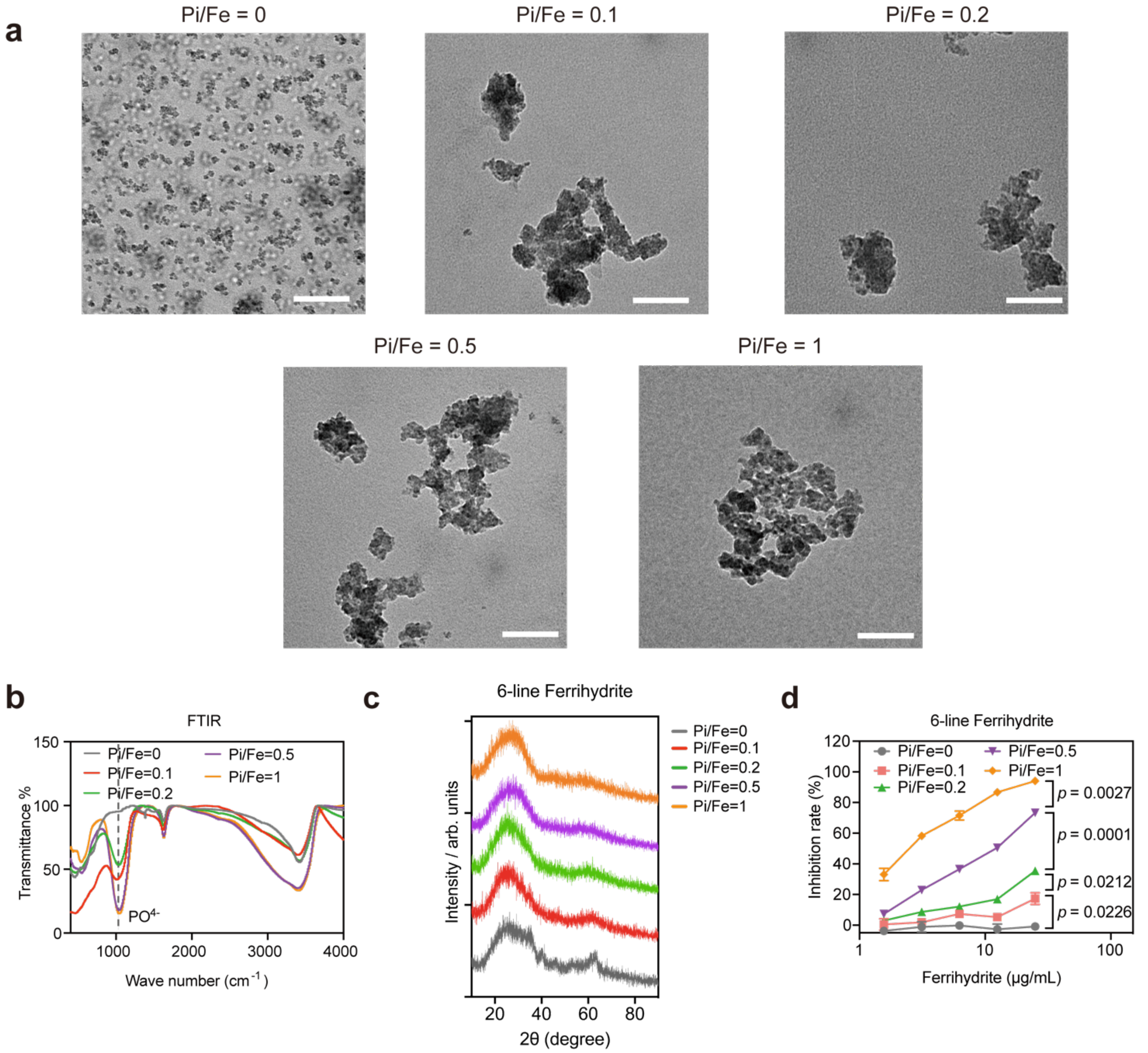

Supplementary Figure 12. Characterization and SOD-like activity of the phosphate-doped 6-line ferrihydrite. (a) TEM images of the 6-line ferrihydrite doped with gradient phosphate; the scale bar referred to 100 nm. (**b**) FTIR spectra of 6-line ferrihydrite doped with gradient phosphate. (**c**) XRD curve of the 6-line ferrihydrite doped with gradient phosphate. (**d**) SOD-like activity of the 6-line ferrihydrite doped with gradient phosphate; data are presented as mean ± SD (n = 3 independent experiments); the significant differences were evaluated by Two-way ANOVA with post-hoc Tukey HSD test. Source data are provided as a Source Data file.

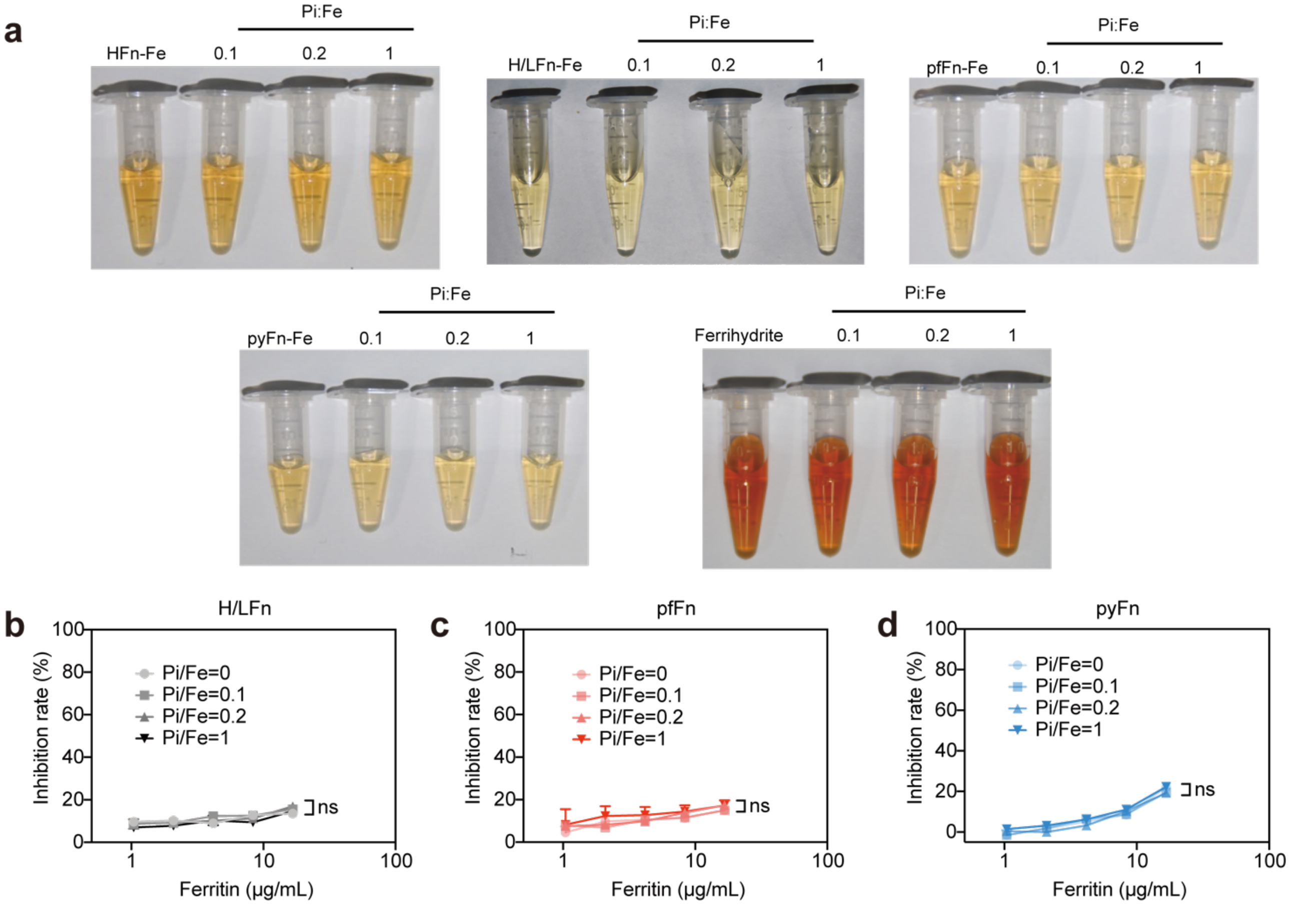

Supplementary Figure 13. Digital images and SOD-like activity of phosphate-absorbed ferritins and 2-line ferrihydrite. (**a**) Digital image of the surface phosphate absorbed ferritins and 2-line ferrihydrite. (**b-d**) SOD-like activity of the surface phosphate absorbed H/LFn, pfFn and pyFn; data are presented as mean ± SD (n = 3 independent experiments); the significant differences were evaluated by Two-way ANOVA with post-hoc Tukey HSD test; p value compared with Pi/Fe=0 of Pi/Fe=0.1, 0.2, 1 in H/LFn group were 0.5129, 0.2377, 0.7936, respectively; in pfFn group were 0.9999, 0.2282, 0.5648, respectively; in pyFn group were 0.193, 0.2216, 0.9855, respectively. Source data are provided as a Source Data file

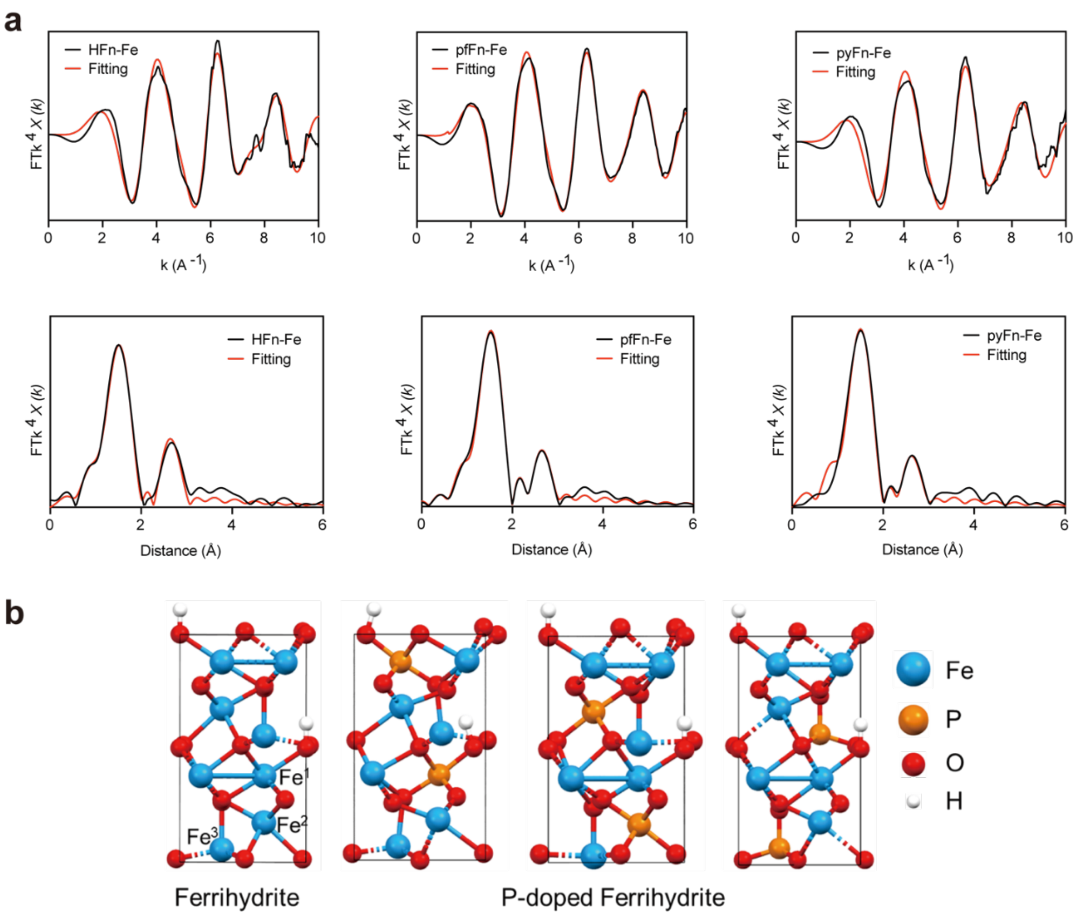

Supplementary Figure 14. Crystal structure of ferrihydrite and FT-XAFS fitting curve of the iron atom in biomineralized ferritins. (**a**) The fitting curve of k-space and R-space of biomineralized HFn-Fe, pfFn-Fe, pyFn-Fe. Source data are provided as a Source Data file. (**b**) Crystal structures of ferrihydrite and P-doped ferrihydrite.

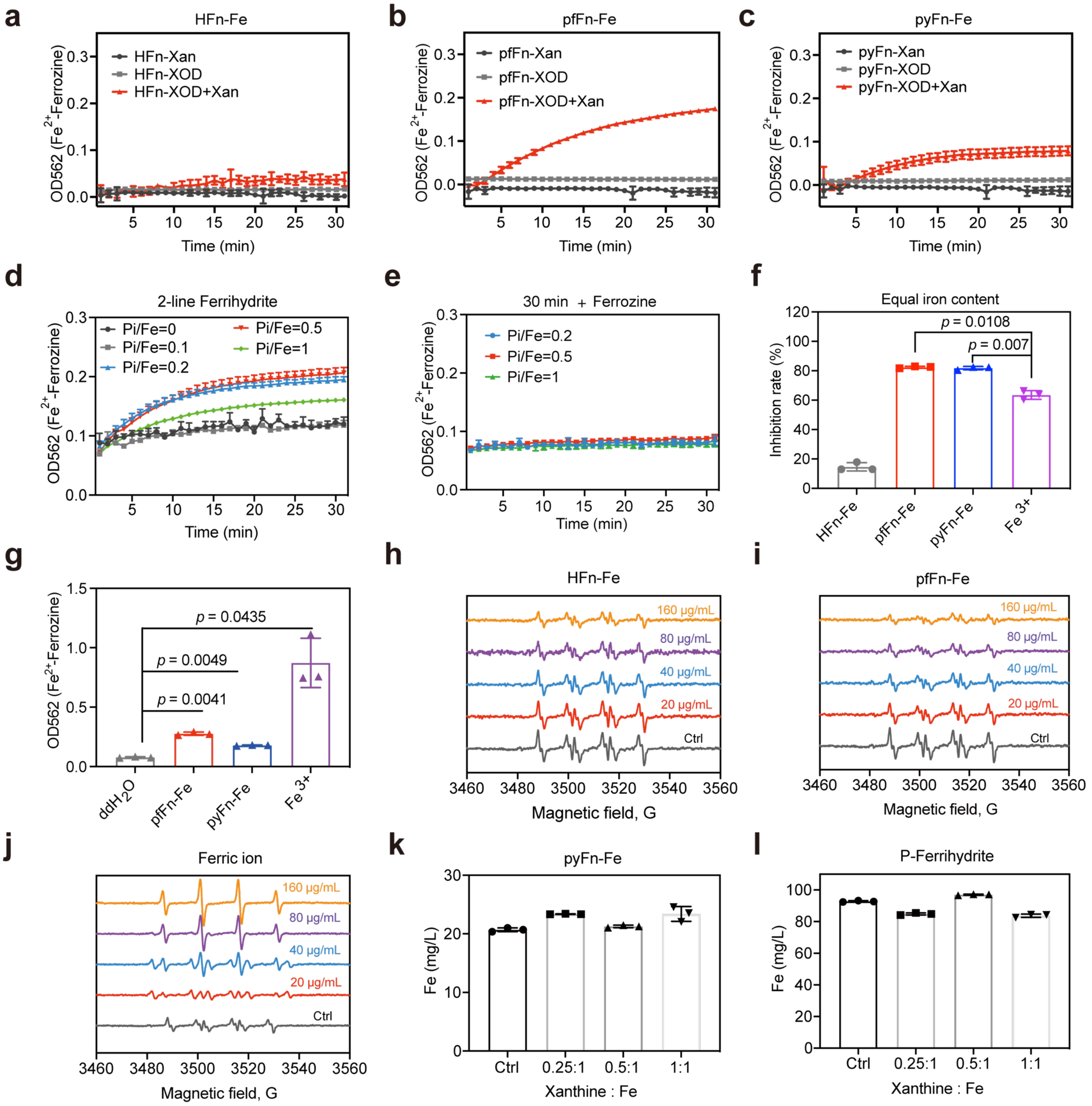

Supplementary Figure 15. Characterization of reaction process of ferritins and phosphate-doped ferrihydrite. (**a-c**) The intermediate ferrous generation of HFn, pfFn, and pyFn in the presence of xanthine, xanthine oxidase and xanthine oxidase & xanthine; data are presented as mean ± SD (n = 3 independent experiments). (**d, e**) The intermediate ferrous generation of phosphate-doped ferrihydrite with the ferrozine added initially or 30 min later; data are presented as mean ± SD (n = 3 independent experiments). (**f**) The SOD-like activities of the ferritins and ferric salt in the same total iron level; data are presented as mean ± SD (n = 3 independent experiments); the significant differences were evaluated by One-way ANOVA with post-hoc Tukey HSD test. (**g**) Ferrous production of the archaeal ferritins and ferric salt in the equal iron content with the ferrozine added initially; data are presented as mean ± SD (n = 3 independent experiments); the significant differences were evaluated by One-way ANOVA with post-hoc Tukey HSD test. (**h, i**) ESR spectrum of the DMPO/HOO• generated by Xan/XOD in the presence of gradient HFn and pfFn. To assess the changes of ROS under consistent

condition, the Ctrl in both **h**, **i**, as well as **Figure 6g** referred to the same Xan/XOD system without ferritins. Marked concentration referred to the iron content of ferritins determined by ICP/MS. (**j**) ESR spectra of DMPO/ HOO• or DMPO/ •OH generated by Xan/XOD in the presence of gradient Fe^{3+}. **(k, l)** Iron content of the biomineralized pyFn and phosphate-doped 2-line ferrihydrite after their reaction with gradient Xanthine; data are presented as mean ± SD (n = 3 independent experiments). Source data are provided as a Source Data file

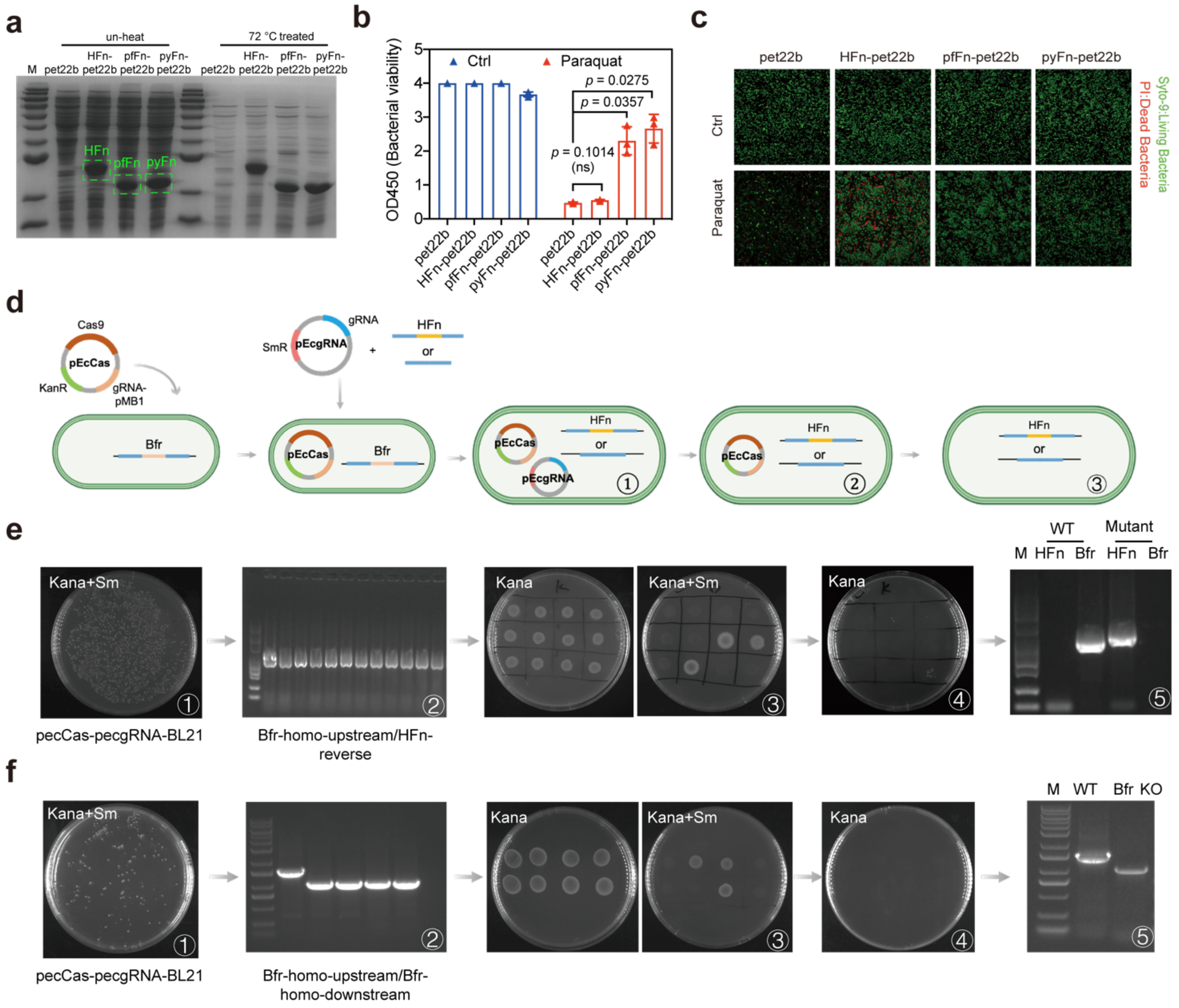

Supplementary Figure 16. Characterization of the ferritin overexpressed BL21(DE3) strain and the process of bacterioferritin gene knockout and substitution. (**a**) SDS-PAGE of ferritin transformed BL21(DE3) strain with or without heat treatment. M represents protein marker. (**b**) The dehydrogenase activities of the ferritin transgenic *E. coli* with or without the paraquat stimulation; data are presented as mean ± SD (n = 3 independent experiments); the significant differences were evaluated by One-way ANOVA with post-hoc Tukey HSD test. (**c**) CLSM image of the living and dead bacteria of transgenic ferritin under the paraquat pressure. (**d**) Scheme of the gene substitution strategy by the CRISPR/Cas9 system. (**e**) The process of the gene substitution: 1. The image of the transgenic strain with the resistance of kanamycin (Kana) and Spectinomycin (Sm). 2. The nucleic acid electrophoresis image of the transgenic BL21 which was cloned by genomic forward primer (Bfr-

homo-upstream) and reverse primer inner the HFn (HFn-inner-reverse). 3. Image of transgenic BL21 after the pecgRNA plasmid was cured. 4. Image of transgenic BL21 after the pecCas plasmid was cured. 5. The Nucleic acid electrophoresis image of the WT and Bfr substituted strain which was cloned by genomic forward primer (Bfr-homo-upstream) and reverse primer inner the HFn or Bfr (HFn-inner-reverse, Bfr-inner-revers). (**f**) The process of the Bfr gene knockout: 1. The image of the transgenic strain with the resistance of kanamycin and spectinomycin. 2. The nucleic acid electrophoresis image of WT and Bfr knockout strain which was cloned by genomic forward primer (Bfr-homo-upstream) and genomic reverse primer (Bfr-homo-downstream). 3. Image of Bfr knockout strain after the pecgRNA plasmid was cured. 4. Image of Bfr knockout strain after the pecCas plasmid was cured. 5. The Nucleic acid electrophoresis image of the WT and Bfr knockout strain which was cloned by genomic forward primer (Bfr-homo-upstream) and genomic reverse primer (Bfr-homo-downstream); M represents DNA marker. Source data are provided as a Source Data file.

Supplementary Tables

Supplementary Table 1. Fe k-edge EXAFS curve Fitting Parameters.

Sample	Shell	N	R(Å)	$\sigma^2(10^{-2}\ Å^2)$	$\Delta E_0(eV)$	r-factor (%)
**Fe foil**	Fe-Fe	12.0	2.54	-	-	-
**HFn-Fe**	Fe-O	6.0	1.99	0.8	-5.1	0.02
	Fe-Fe	2.8	3.04	1.3	-5.4	
**pfFn-Fe**	Fe-O	6.0	1.99	0.7	-3.0	0.01
	Fe-P	0.7	2.65	1.3	5.2	
	Fe-Fe	2.2	3.03	1.3	-6.6	
**pyFn-Fe**	Fe-O	6.0	1.98	0.7	-6.3	0.01
	Fe-P	0.6	2.69	1.3	-4.1	
	Fe-Fe	1.6	3.06	1.3	-3.1	

[a]N is the coordination number for the absorber-backscatter pair, R is the average absorber-backscatter distance, σ^2 is the Debye-Waller factor, and ΔE_0 the inner potential correction. The accuracies of above parameters are estimated as CN, ±20%; R, ±10%; σ^2, ±20%; ΔE_0, ±20%. The data range used for data fitting in k-space (Δ k) and R-space (Δ R) are 3.0-10.0 $Å^{-1}$ and 1.0-3.0 Å, respectively.

Supplementary Table 2. Plasmids and primers used in the CRISPR/Cas9 system.

Plasmid/Primer names	sequence	referee
pecCas	From Genescript (Nanjing, China)	1
pecgRNA	Modified from pecgRNA (Genescript Nanjing, China)	1
HFn-homo-pet22b (containing HFn and homologous arms)	Constructed in our lab	
Bfr-homo-pet22b (containing BFR homologous arms)	Constructed in our lab	
Bfr-gRNA-forward	tagttggcgcgaagaatttgcgtg	
Bfr-gRNA-reverse	aaaccacgcaaattcttcgcgcca	
Bfr-homo-upstream	gggcggcactaacctgtctc	
Bfr-homo-downstream	gttgctgctgctatcgcctg	
HFn-inner-reverse	acaggtattccgccagaccagat	
Bfr-inner-reverse	agttccgtttccagccagtc	

Supplementary References

1. Li Q, Sun B, Chen J, Zhang Y, Jiang Y, Yang S. A modified pCas/pTargetF system for CRISPR-Cas9-assisted genome editing in Escherichia coli. *Acta Biochim Biophys Sin (Shanghai)* **53**, 620-627 (2021).
