# Supplementary material for: A natural biogenic nanozyme for scavenging superoxide radicals
Source: Nat Commun. 2024 Jan 3;15:233. doi: 10.1038/s41467-023-44463-w (PMC10764798; doi:10.1038/s41467-023-44463-w)
Supplement: Supplementary file 3 — Reporting Summary [file 41467_2023_44463_MOESM3_ESM.pdf]

nature portfolio

Corresponding author(s): Kelong Fan

Last updated by author(s): Nov 23, 2023

Reporting Summary

Nature Portfolio wishes to improve the reproducibility of the work that we publish. This form provides structure for consistency and transparency in reporting. For further information on Nature Portfolio policies, see our Editorial Policies and the Editorial Policy Checklist.

Statistics

For all statistical analyses, confirm that the following items are present in the figure legend, table legend, main text, or Methods section.

n/a	Confirmed	
☐	☒	The exact sample size (n) for each experimental group/condition, given as a discrete number and unit of measurement
☐	☒	A statement on whether measurements were taken from distinct samples or whether the same sample was measured repeatedly
☐	☒	The statistical test(s) used AND whether they are one- or two-sided<br>*Only common tests should be described solely by name; describe more complex techniques in the Methods section.*
☒	☐	A description of all covariates tested
☐	☒	A description of any assumptions or corrections, such as tests of normality and adjustment for multiple comparisons
☐	☒	A full description of the statistical parameters including central tendency (e.g. means) or other basic estimates (e.g. regression coefficient) AND variation (e.g. standard deviation) or associated estimates of uncertainty (e.g. confidence intervals)
☐	☒	For null hypothesis testing, the test statistic (e.g. F, t, r) with confidence intervals, effect sizes, degrees of freedom and P value noted<br>*Give P values as exact values whenever suitable.*
☒	☐	For Bayesian analysis, information on the choice of priors and Markov chain Monte Carlo settings
☒	☐	For hierarchical and complex designs, identification of the appropriate level for tests and full reporting of outcomes
☒	☐	Estimates of effect sizes (e.g. Cohen's d, Pearson's r), indicating how they were calculated

Our web collection on statistics for biologists contains articles on many of the points above.

Software and code

Policy information about availability of computer code

Data collection	Dynamics Light Scattering spectra were obtained by Wyatt Technology 271-DPN (USA). Inductively coupled plasma mass spectrometry results were obtained by Agilent ICPMS7800 (USA). Transmission electron microscopy (TEM) images were obtained by using FEI Tecnai G2 F30 (FEI, USA). Powder X-ray diffraction (XRD) data were collected by using Bruker D8 ADVANCE (Germany) with a scan rate of 4 ° / min. FT-IR spectra were recorded by Nicolet IS10 (USA). X-ray photoelectron spectroscopy (XPS) spectra were recorded by Thermo Escalab 250Xi (USA). Electron spin resonance (ESR) spectra were recorded by Bruker A300-9.5/12 (Switzerland) at room temperature. Confocal laser scanning microscopy images were obtained by ZIESS-LSM700 (Germany). Flow cytometry data were collected by FACS CaliburTM, Becton Dickinson (USA). Absorbance was measured using SpectraMax® M4 Microplate Reader (USA). Fe-K edge XAFS spectra were obtained on the beamline 1W1B of Beijing Synchrotron Radiation Facility by transmission mode(China).
Data analysis	Data were analyzed by Pymol(2.3.4), Image J 1.52(100), Origin 2021, XPSPEAK41, ZEN 2010, FlowJo 10.4 and Graphpad prism 8.

For manuscripts utilizing custom algorithms or software that are central to the research but not yet described in published literature, software must be made available to editors and reviewers. We strongly encourage code deposition in a community repository (e.g. GitHub). See the Nature Portfolio guidelines for submitting code & software for further information.

Data

Policy information about availability of data

All manuscripts must include a data availability statement. This statement should provide the following information, where applicable:

- Accession codes, unique identifiers, or web links for publicly available datasets
- A description of any restrictions on data availability
- For clinical datasets or third party data, please ensure that the statement adheres to our policy

The data supporting the findings of this study are available from the corresponding authors upon request. All the PDB Structures are available on the PDB database (https://www.rcsb.org/). Source data for the figures and supplementary figures are provided as a Source Data file. Source data are provided with this paper.

Research involving human participants, their data, or biological material

Policy information about studies with human participants or human data. See also policy information about sex, gender (identity/presentation), and sexual orientation and race, ethnicity and racism.

Reporting on sex and gender	N/A
Reporting on race, ethnicity, or other socially relevant groupings	N/A
Population characteristics	N/A
Recruitment	N/A
Ethics oversight	N/A

Note that full information on the approval of the study protocol must also be provided in the manuscript.

Field-specific reporting

Please select the one below that is the best fit for your research. If you are not sure, read the appropriate sections before making your selection.

- [x] Life sciences
- [ ] Behavioural & social sciences
- [ ] Ecological, evolutionary & environmental sciences

For a reference copy of the document with all sections, see nature.com/documents/nr-reporting-summary-flat.pdf

Life sciences study design

All studies must disclose on these points even when the disclosure is negative.

Sample size	No statistical methods were applied to predetermine sample sizes. Sample sizes were determined empirically from previous experimental experience with similar assays or from sizes generally employed in the field. For protein purification, at least three independent different expression bacteria were used to acquire the biological replicates, and the samples were acquired in different days. For in vitro synthesis and activity test, each group involved three independent synthesis or detect processes.
Data exclusions	No data was excluded from the analysis.
Replication	Independent replicates of at least 3 times were used for all experiments. We confirmed that the attempts at replication were successful. Each figure contains detailed independent experimental replicates in the figure.
Randomization	All samples were randomly allocated into experimental groups.
Blinding	All the characterization, enzyme activity test or bacteria experiment were conducted with the sample random grouped, and the investigators were blinded during data collection and analysis.

Reporting for specific materials, systems and methods

We require information from authors about some types of materials, experimental systems and methods used in many studies. Here, indicate whether each material, system or method listed is relevant to your study. If you are not sure if a list item applies to your research, read the appropriate section before selecting a response.

Materials & experimental systems

n/a	Involved in the study
☒	☐ Antibodies
☒	☐ Eukaryotic cell lines
☒	☐ Palaeontology and archaeology
☒	☐ Animals and other organisms
☒	☐ Clinical data
☒	☐ Dual use research of concern
☒	☐ Plants

Methods

n/a	Involved in the study
☒	☐ ChIP-seq
☐	☒ Flow cytometry
☒	☐ MRI-based neuroimaging

Flow Cytometry

Plots

Confirm that:

☒ The axis labels state the marker and fluorochrome used (e.g. CD4-FITC).

☒ The axis scales are clearly visible. Include numbers along axes only for bottom left plot of group (a 'group' is an analysis of identical markers).

☒ All plots are contour plots with outliers or pseudocolor plots.

☒ A numerical value for number of cells or percentage (with statistics) is provided.

Methodology

Sample preparation	For ROS level detection in bacteria, the overnight cultured BL21(DE3) strains with different ferritin plasmids were transformed to the fresh LB medium containing 100 μg/mL ampicillin until the OD600 reached 0.2, then 1 mM IPTG and 1 mM paraquat were simutaneously added to the medium and the inoculums were further incubated at 37 °C, 200 rpm for 12 h. After the treatment, the bacteria were harvested and washed three times by PBS buffer. Subsequently, the DCFH-DA was added to resuspended bacteria in the final concentration of 10 μM and incubated at 37 °C for further 30 min. Then the samples were washed three times by PBS. Finally, the fluorescence intensities were examined using flow cytometry.
Instrument	FACS CaliburTM, Becton Dickinson, Franklin Lakes, NJ, USA
Software	FlowJo 10.4
Cell population abundance	We collected 30000 cells per samples for test.
Gating strategy	Cells were gated according size and granularity of forward and side scatter (SSC and FCS) and the cell gate was analyzed for specific fluorescence.

☒ Tick this box to confirm that a figure exemplifying the gating strategy is provided in the Supplementary Information.
